# Pharmacological Mobilization and Recruitment of Stem Cells in Rats Stops Abdominal Adhesions After Laparotomy

**DOI:** 10.1038/s41598-019-43734-1

**Published:** 2019-05-09

**Authors:** Kenichi Iwasaki, Ali Reza Ahmadi, Le Qi, Melissa Chen, Wei Wang, Kenji Katsumata, Akihiko Tsuchida, James Burdick, Andrew M. Cameron, Zhaoli Sun

**Affiliations:** 1 Department of Surgery, The Johns Hopkins University School of Medicine, Baltimore, MD USA; 2 Department of Gastrointestinal Surgery, Tokyo Medical University, Shinjuku-ku, Tokyo Japan

**Keywords:** Biologics, Preclinical research

## Abstract

Adhesions are a very common complication in the abdominal surgery. Animal studies and human trials have evaluated strategies designed to reduce and prevent postsurgical adhesions but few have an evidence base that justifies routine use. A strategy to prevent adhesions effectively remains an urgent need. We studied a reproducible model of intra-peritoneal adhesion formation in rats using laparotomy with several peritoneal sutures to produce the adhesions. Here we show that entraining endogenous stem cells into injury sites using the combined effect of AMD3100 and low-dose FK-506 (AF) can reduce the adhesion score significantly and abolish peritoneal adhesions in 45% of animals in a rat model of severe postsurgical intra-abdominal adhesions, compared with saline controls. Searching for mechanisms, we found AF treatment dramatically increased SDF-1 expressing cells, HGF expressing Ym1+ M2 macrophages and CD133+ stem cells in the injury sites of peritoneal surface at day 5 post-operation. Our results demonstrate that medically induced recruitment of autologous stem cells using AF significantly reduced postsurgical intra-abdominal adhesions. These findings suggest a novel effective therapeutic approach to preventing adhesions in patients.

## Introduction

Formation of peritoneal adhesions which are a normal response to injury of the peritoneal surfaces aimed at repairing the damage following surgery that violates the peritoneum has become frequent^1^. These adhesions commonly cause problems and require additional procedures^1–5^. Up to 20% of these patients will experience symptoms ranging from pain to intestinal obstruction^2^. Approximately 10% of bowel obstructions caused by adhesions require reoperation known as adhesiolysis^1^, and the presence of severe adhesions at reoperation leads to increasing surgical difficulties and longer surgical times^1,3,4^. Pelvic peritoneal adhesions are responsible for approximately 10 percent of cases of female infertility^6^. Although attempts at amelioration have been adopted, the number of adhesiolysis procedures has been growing over the past decade^5^. The need for this is not only unfortunate for patients but increases the cost of health care.

Peritoneal injury produces reaction aimed at healing the damaged peritoneal surfaces which results in the formation of adhesions (fibrotic scars) between two damaged peritoneal surfaces. In the last few decades attempts to stop this have included improved surgical techniques, optimized laparoscopy conditions, anti-inflammatory pharmacotherapies targeted at the inflammatory response and/or fibrin deposition, and creating a material interposition for prevention of peritoneal apposition. Sodium hyaluronate/carboxymethylcellulose (Seprafilm), oxidized regenerated celluose (Intercreed) and 4% icodextrin solution (Adept) are approved and generally viewed as best practice to prevent adhesions^7–11^. Nevertheless, these have been only marginally helpful and a strategy to prevent adhesions effectively remains an urgent need.

We here report a new solution. This is from a novel entrainment of bone marrow precursor cells using a combination of AMD3100 (plerixafor) and FK506 (tacrolimus) that was found by chance to enable permanent liver allograft protection from rejection and survival in rats with just one week of treatment^12^. Further, treatment for a week every month for three months provided the same protection for rat and swine renal allografts^13,14^. This protection against rejection involved the presence of recipient stem cells found in the graft (allograft chimerism) causing local immunosuppression. We also showed that this medical mobilization of undifferentiated bone marrow cells produced a 25% reduction in healing time of skin wounds^15^. Importantly, this allowed reduced scars and provided for hair follicle growth not seen in the damaged area in controls. In the organ transplantation models, we incidentally observed that there were fewer abdominal peritoneal adhesions in small animals and no adhesion formation in large animals treated with AF combination drugs, even 3–4 years after pig kidney transplantation, however animals treated with saline or single drug formed severe intra-abdominal adhesions. In experiments with 85% partial hepatectomy it was noted that adhesions were minimized in the test group receiving combination therapy^16^. Based on these observations, we hypothesized that medical conscription and recruitment of undifferentiated bone marrow cells by this drug combination may promote regeneration of damaged peritoneal surfaces following surgery, and therefore prevents the formation of peritoneal adhesions. Here we test this hypothesis objectively by testing it in severe peritoneal scarring in rats. We show that this treatment was able to reduce the adhesion score significantly and abolish peritoneal adhesions in 45% of animals.

## Results

### Creating suture knots on the parietal peritoneum recapitulates postsurgical intra-abdominal adhesion in rats

**Figure 1:**
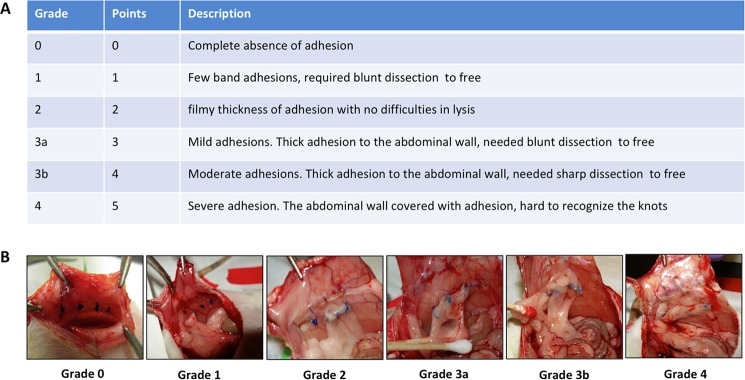
Adhesion grade and assessment in a rat model of postsurgical intra-abdominal adhesion. Abdominal adhesions were induced by creating four 1–0 Prolene suture knots on the parietal peritoneum in a linear distribution. Each suture encapsulated approximately 2 cm of the parietal peritoneum. The diameter of the tied suture was approximately 5 mm. (**A**) Scoring criteria of adhesion grades and points. (**B**) Representative images of the intra-abdominal adhesion formation.

All animals that underwent surgery survived, no wound complications or infections occurred and all animals completed the study protocol. The surgical procedure reliably produced abdominal adhesions in control animals 14 days post-operation. Based on the adhesion grade and assessment criteria (Fig. 1), a majority of animals (10/12) in the control group with this surgical procedure developed severe intra-abdominal adhesions.

### AF combination therapy, but not single drug prevents postsurgical abdominal adhesions in rats

**Figure 2:**
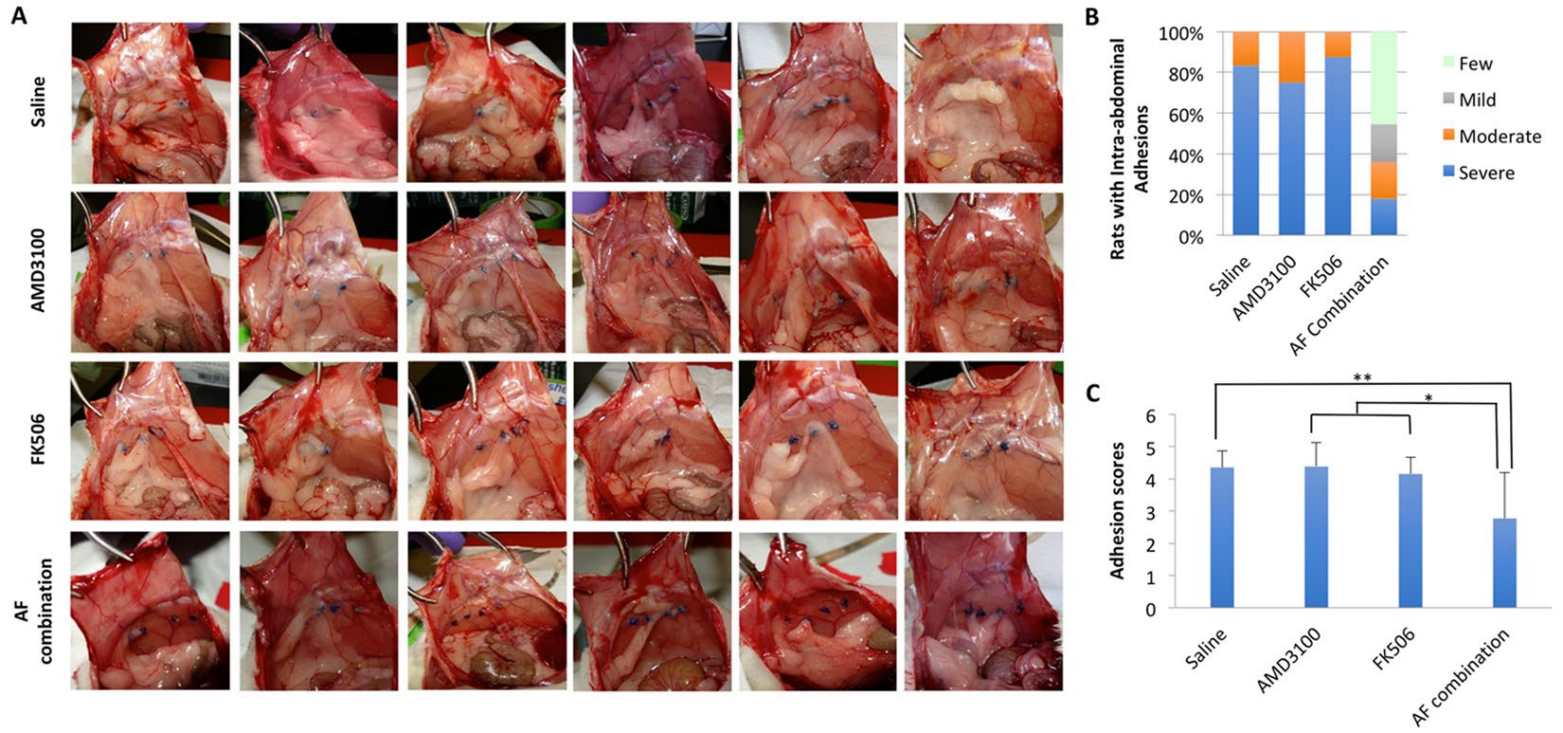
Analysis for postsurgical adhesion grades for each group. (**A**) Representative images of the intra-abdominal adhesion formation from six animals in each treatment group on day 14 post operation. (**B**) Percentage of rats with few, mild, moderate or severe postsurgical intra-abdominal adhesions in different treatment groups. (**C**) Quantitative analysis of the intra-abdominal adhesion scores in animals treated with saline, single drug or AF combination. The adhesion scores remained the same in animals receiving AMD3100 (4.38 ± 0.74, n = 8) or FK506 (4.15 ± 0.52, n = 8) alone treatment compared to control animals treated with saline (4.35 ± 0.52, n = 12). The adhesion scores were significantly decreased in dual drug treated animals (2.77 ± 1.42, n = 11) compared to animals treated with saline or single drug. The data are shown as mean ± standard deviation (SD). *P < 0.05, **P < 0.01.

On post-operative day 14, 83.3% animals (10/12) that received saline control treatment subcutaneously developed severe intra-abdominal adhesions (adhesion scores: >4), while 16.7% (2/12) showed moderate intra-abdominal adhesions (adhesion scores: =3–4). Animals that received AMD3100 sc displayed severe (75%; 6/8) or moderate (25%; 2/8) intra-abdominal adhesions. Similarly, seven out of eight animals (87.5%) that received sc FK506 developed severe intra-abdominal adhesions, only one animal showed mild intra-abdominal adhesion (adhesion scores: =2–3). In contrast, animals that received a combination of AMD3100 and FK506 (AF) therapy sc displayed significantly less intra-abdominal adhesion formation (Fig. 2A): about 45% animals (5/11) showed few intra-abdominal adhesions, 18% animals with mild intra-abdominal adhesions (2/11) (adhesion scores: <2), 18% (2/11) animals developed moderate intra-abdominal adhesions and only 18% animals had severe intra-abdominal adhesions (Fig. 2B). The intra-abdominal adhesion scores were further analyzed quantitatively in animals treated with saline, single drug or AF combination. The adhesion scores remained the same in animals receiving AMD3100 (4.38 ± 0.74, n = 8) or FK506 (4.15 ± 0.52, n = 8) alone compared to control animals treated with saline (4.35 ± 0.52, n = 12). However, the adhesion scores were significantly decreased in dual drug treated animals (2.77 ± 1.42, n = 11) compared to saline or single drug treated animals (Fig. 2C).

### Entrainment of CD133 cells into the injury of the peritoneal surface parallels elevated SDF-1 and HGF following therapy

**Figure 3:**
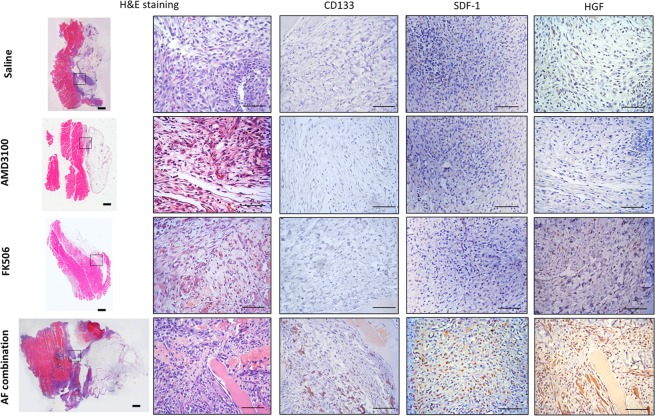
Histological analysis of the intra-abdominal adhesion tissues. Hematoxylinand eosin (HE) staining and a representative result in intra-abdominal adhesion tissues of immunohistochemical staining for CD133, SDF-1 and HGF. Few CD133, SDF-1 or HGF positive cells were recognized in adhesion tissues from saline control or single drug treated animals on day 5 after surgery. In contrast, numerous CD133, SDF-1 and HGF positive cells were recognized in adhesion tissues from AF combination treated animals on day 5 after surgery. Images were taken at 400× magnification. Scale bar = 100 um.

We have reported a synergy between AMD3100 and low-dose FK506 for entrainment of bone marrow-derived CD133 stem cells into damaged tissue^12–16^. To find whether this treatment recruits undifferentiated bone marrow cells into the injury sites of the peritoneal surface after creating suture knots on the parietal peritoneum, immunohistochemistry staining for CD133, SDF-1 and HGF was done. Figure 3 demonstrates that few CD133 positive cells were evident in intra-abdominal adhesion tissues from saline control or single drug treated animals on day 5 following surgery. In contrast, numerous CD133 positive cells were found in intra-abdominal adhesion tissues from AF combination treated animals on day 5 after surgery.

SDF1 (stromal cell-derived factor-1) can entrain undifferentiated stem cells into injured tissues^17,18^ by its attraction to the CXCR4 receptor on mobilized stem cells. Few SDF-1 positive cells were recognized histologically in animals treated with saline or single drug on day 5 after surgery. However, SDF-1 positive cells were strongly increased in treated animals (Fig. 3). Importantly, the higher number of SDF-1 positive cells in intra-abdominal adhesion tissues of AF treated animals paralleled the increase in CD133 stem cells.

HGF is crucial in regeneration, cellular growth and motility, and tissue formation. It has been reported that HGF prevents postoperative peritoneal adhesions^19^, probably through stimulating the regeneration of peritoneal mesothelial cells^20^, inhibition of collagen deposition and its fibrinolytic capacity^21^. No HGF expression was found in adhesions from control animals treated with saline or AMD3100, but a few HGF positive cells were present in animals receiving low-dose FK506 alone on postoperative day 5. The number of HGF positive cells was dramatically increased in animals treated with AF combination therapy on postoperative day 5. Thus, entrainment of CD133 cells in the injury sites on the peritoneal surface was associated with increased HGF expression. The increased HGF expression may be important in preventing adhesions.

### AF combination therapy increases HGF-producing M2 macrophages in the injury sites of peritoneal surface

**Figure 4:**
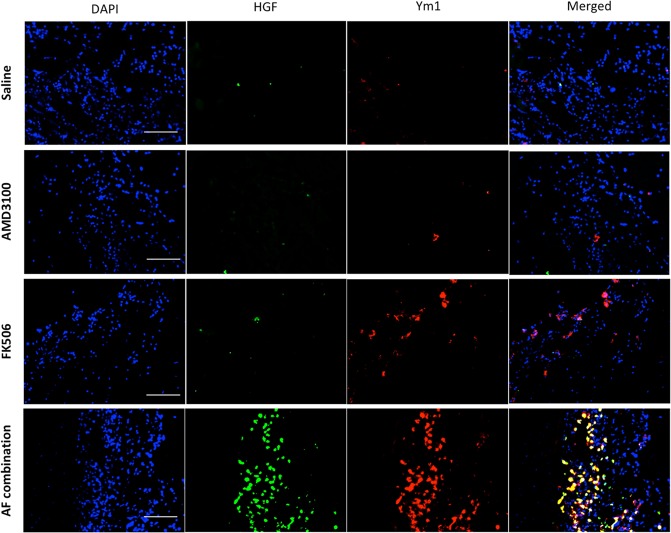
Immunofluorescence double staining for HGF (green) and Ym1 (red) in the intra-abdominal adhesion tissues. Tissue sections were counter-stained with DAPI (blue). HGF and Ym1 (a marker of M2 macrophages) positive cells increased on day 5 after surgery in adhesion tissues from AF treated animals, and double positive cells were recognized. Images were taken at 400× magnification.

To determine whether AF combination therapy promotes immunomodulatory M2-polarization^22^ of infiltrating macrophages in the injury sites of the peritoneal surface, immunofluorescent staining for the known M2 macrophage marker Ym1/Chi3l3 was performed. Few Ym1 positive cells were recognized in intra-abdominal adhesion tissues from saline control or AMD3100 treated animals, while Ym1 positive cells were slightly increased in low-dose FK506 treated animals on day 5 following surgery. In contrast, Ym1 positive cells were dramatically increased in adhesion tissues from animals with AF combination therapy (Fig. 4). Interestingly, a majority of Ym1 positive cells also expressed HGF in combination-treated animals but not those treated with FK506 alone. These results suggest that low-dose FK506 alone may promote M2 polarization of infiltrating macrophages, while the combination resulted in synergistic facilitation of M2 polarization and entrainment of M2 macrophages. Production of HGF by infiltrating M2 macrophages may not only promote healing of the injured peritoneal surface but also contribute to freedom from peritoneal scarring.

## Discussion

These findings show the importance of endogenous bone marrow-derived stem cells in preventing postsurgical adhesions. In control animals treated with stem cell mobilizer AMD3100 or low dose FK506 alone, few stem cells appeared in the peritoneal injury and this injury resulted in formation of severe intra-abdominal adhesions following surgery. AF combination therapy dramatically increased CD133 and HGF expressing M2 macrophages in the injured peritoneum. We have shown that this was associated with attenuation of intra-abdominal adhesions.

The peritoneum lines the abdomen as a protective film of mesothelium that reduces friction between the viscera^23^. The peritoneum is easily damaged due to the loose association between the mesothelial cells^24^. Peritoneal injury results in tissue ischemia^25–27^, which sets in motion the reparative hypoxia/coagulation cascade. But this increased oxidative stress leads to mesothelial cell damage and intra-abdominal adhesion formation^28^. In this study, we have established a postsurgical intra-abdominal adhesion model in rats by creating four 1–0 Prolene suture knots on the parietal peritoneum in a linear distribution. The suture knots caused local tissue ischemia that is essential for triggering adhesion formation^28^. Indeed, most control animals (83%) developed severe intra-abdominal adhesions on postoperative day 14 (Fig. 2B). These results indicate that this suture knots-induced adhesion technique is a reliable model of severe postsurgical intra-abdominal adhesions that is suitable for testing therapeutic interventions.

Because adhesions occur at the injured surface lining of the peritoneum, a rapid rate of remesothelialization and restriction of inflammation likely are important factors that limit postoperative adhesion development. The healing time of small and large peritoneal wounds is the same^29^ suggesting that healing is not a local event at the margins of the injury and that stem cells may be involved. Mesothelial stem cells can differentiate into mesothelial cells which can derive from differentiation of adult stem cells in adjacent muscle and contribute to healing^29,30^. Intraperitoneal delivery of autologous stem cells from muscle prevents abdominal adhesions^29^. Interestingly, intra-abdominal application of mesenchymal stem cells (MSCs) showed promising results for preventing postsurgical intra-abdominal adhesions^31–33^. This provides a precedent for prevention of abdominal adhesions by other adult stem cells. Because the *in vitro* preparation of autogenous stem/progenitor cells takes time and raises questions of quality, quantity and effectiveness, this stem cell therapy has limited practical application in the treatment of postsurgical abdominal adhesions. For this reason, the mobilization and recruitment of autologous stem cells that we have described is a new paradigm for a simple way to provide stem cells to injured sites of the peritoneum.

**Figure 5:**
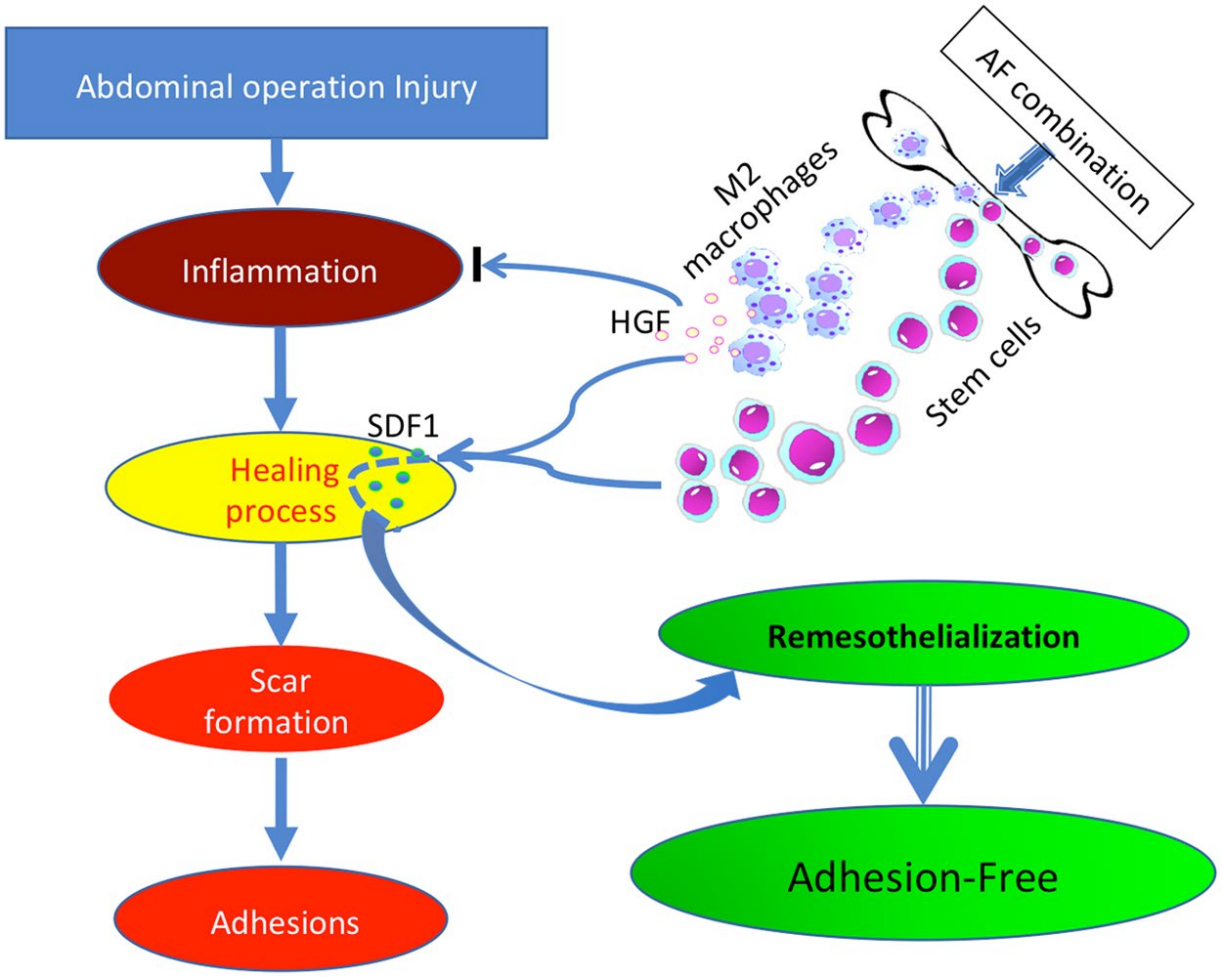
Schematic representation of therapeutic mechanism of AF combination treatment in preventing postsurgical intra-abdominal adhesion. AMD3100 and low-dose FJ506 reacted synergistically in mobilization and recruitment of bone marrow-derived CD133 stem cells. Low-dose FK506 promotes M2 polarization of infiltrating macrophages and induces HGF production. AF combination therapy may prevent/attenuate postsurgical intra-abdominal adhesion formation via targeting two important factors – remesothelialization and inflammation through recruiting CD133 stem cells and HGF producing M2 macrophages. Rapid remesothelialization following injury may result in adhesion free healing.

The current studies are based on our previous findings with allograft tolerance^12–16^. The mechanism of action of the combination treatment is that it mobilizes bone marrow stem cells into the circulation via blocking the CXCR4 binding with SDF-1, but also promotes accumulation of the stem cells into the injured organ/tissues as the AMD3100 CXCR4 blocking effect wanes (Fig. 5). In our experiments, neither agent alone increased stem cells in the injured sites of peritoneum on post-operation day 5, although Ym1 positive M2 macrophages were slightly increased in low-dose FK506 treated animals. Consequently, single drug treatment did not reduce postsurgical abdominal adhesion formation. In contrast, the AF combination treatment dramatically increased CD133 cells and M2 macrophages in the injured sites of peritoneum and subsequently prevented/attenuated postsurgical intra-abdominal adhesion formation. Bone marrow-derived stem cells including MSCs have two unique properties: the ability to differentiate into the tissue type requiring regeneration; and their immunomodulatory properties that reduce inflammation and fibrosis through cytokine/growth factor release. Thus, concentrated CD133 stem cells in the injured peritoneal areas may not only promote remesothelialization, but also reduce the inflammatory response.

Postsurgical adhesion formation is due to the peritoneal injury plus the associated inflammation. The inflammatory cells are predominantly macrophages at 24 hours following injury^34^. M2 macrophages secret anti-inflammatory cytokines (IL-4, IL-10) which inhibit production of pro-inflammatory cytokines (e.g. IL-1, 6 and TNF). It was found that increased M2 macrophages in sites of polyglycolic acid injury correlated with fewer peritoneal adhesions^35^. By using immunofluorescence staining for Ym1 (Chi3l3), a marker of M2 macrophages, we found that AF combination therapy dramatically increased Ym1 positive M2 macrophages in the injured peritoneum. Interestingly, a majority of Ym1 positive cells co-stained with hepatocyte growth factor (HGF) which is important in the action of M2 macrophages in intestinal healing through the secretion of HGF^36^. HGF decreases adhesion formation by inhibiting IFN-γ and PAI-1^37^ and induces mesothelial cell proliferation^38^. Thus, HGF-producing M2 macrophages induced by AF combination therapy may reduce adhesion formation through regulating the inflammatory response and promoting wound repair/regeneration.

In our experiments, low-dose FK506 increased Ym1 positive M2 macrophages in the injured peritoneum. Interestingly, low-dose FK506 had a synergistic effect with AMD3100 in increasing M2 macrophages in the injured sites, similar to the report from Bai *et al*.^39^. FK506 at low dose activates the bone morphogenetic protein (BMP) signaling pathway^40^ through FKBP12 ligands^41^ and BMP4 expression induces macrophage polarization towards M2^42^. In addition, MSCs can induce M2-like macrophages *in vitro* that can suppress T cell and NK cell immune response and induce regulatory T cells^43,44^. CD133 is also a marker of circulating MSCs^45^. A high concentration of CD133 stem cells in the injured peritoneal sites may create an environment conducive to M2 macrophages.

Evidence from bone marrow transplant recipients suggests that the bone marrow progenitor cells might regenerate mesothelium^46^. Although mobilized bone marrow stem cells may generate mesothelial cells and promote remesothelialization, we chose to direct our focus to attenuation of intra-abdominal adhesion formation. Unlike reports of the effect of delivering processed adult stem cells into the peritoneal cavity in ameliorating intra-abdominal adhesion, this study expands on that to demonstrate that pharmacological mobilization and entrainment of the stem cells can reduce/attenuate postsurgical abdominal adhesion formation by stem cells accumulated directly from the circulation through release from the bone marrow. Our AF combination therapy may prevent/attenuate postsurgical intra-abdominal adhesion formation via targeting two important factors – remesothelialization and inflammation through recruiting CD133 stem cells and HGF producing M2 macrophages.

This effect of warding off adhesive peritoneal healing is not incompatible with the increase in speed and completeness of healing of all three intestinal layers in colonic anastomoses that we also found with this treatment (Chen *et al*. unpublished data). In both cases this combination treatment optimized the ability of the stem cells to do the same thing: return the damaged tissue to normal. That required normal mesothelium for the peritoneal surface and normal bowel in the case of the experiments with intestinal anastomoses (and no adhesions to the suture line). This predisposition to return inflamed or damaged tissue to normal is likely a generally applicable feature of this combination therapy.

## Materials and Methods

### Animals

Fifty-five Lewis rats including fourteen males and forty-two females were purchased from Hilltop Lab Animals Inc. (Scottdale, PA, USA) and used at 12 to 15 weeks of ages with body weight between 250 to 350 grams. Animals were maintained in a pathogen-free facility of Johns Hopkins University School of Medicine and all animal experiments were performed in accordance with the United States National Institutes of Health (NIH) guidance^12,13,15^. All animal protocols were reviewed and approved by the Johns Hopkins University Animal Care and Use Committee.

### A model of postsurgical intra-abdominal adhesions

Animals were anesthetized with the inhalation of Isoflurane (Baxter, IL, USA) and the abdominal skin was prepared and disinfected by povidone-iodine before the procedure^12,13^. A 3 cm vertical midline incision was made to access the abdominal cavity. Abdominal adhesions were induced by creating four 1–0 Prolene (Ethicon, Blue Monofilament) suture knots on the anterior parietal peritoneum in a linear distribution. Each suture encapsulated approximately 2 cm of the parietal peritoneum. The diameter of the tied suture was approximately 5 mm. After the creation of the sutures, the peritoneum was closed in two layers by interrupted 3–0 silk sutures. All of the surgical procedures were performed by the same surgeon.

### Experimental design

Fifty-five animals with surgical procedures were allocated into four experimental groups: (1) Control group (n = 16): animals received the same volume of saline (2 ml/kg, subcutaneous injection) immediately after surgery and every other day for 10 days; (2) AMD3100 treatment group (n = 12): animals received AMD3100 (1 mg/kg, subcutaneous injection) immediately after surgery and every other day for 10 days; (3) FK506 treatment group (n = 12): animals received FK506 (0.1 mg/kg, subcutaneous injection) immediately after surgery and every other day for 10 days; and (4) AF treatment group (n = 15): animals received AF combination therapy (AMD3100 1 mg/kg and FK506 0.1 mg/kg, subcutaneous injection) immediately after surgery and every other day for 10 days. Sixteen animals (n = 4/group) were sacrificed for collecting tissue samples on postoperative day 5, and the other animals were sacrificed on postoperative day 14 for evaluation of intra-abdominal peritoneal adhesions.

### Adhesion grade and assessment

On postoperative day 14 rats were euthanized and a U-shape incision to avoid adhesion sites was used for determination of abdominal adhesions. Adhesion sites were photographed and the difficulty to lyse the adhesion was described by a surgeon without knowing the treatment. The severity of intra-abdominal adhesions was evaluated and scored blindly on the pictures by five individual surgeons who were not involved in surgery and treatment to control for bias. For detailed assessment, the scoring criteria of Zühlke *et al*.^47^ was modified by including evaluation of the difficulty to lyse the adhesion which is described in the classification of Mazuji *et al*.^48^ (Fig. 1A). The adhesion scores (mean ± SD) were calculated for each animal to minimize inter-observer variability. Those scores were then used to calculate mean ± SD adhesion scores for each treatment group.

### Histological analysis

The parietal peritoneum and surrounding adhesion tissues were explanted and fixed in 2% PFA. Cut sections of 6 µm were prepared from PFA-fixed paraffin-embedded tissues for CD133, SDF-1 or HGF staining or frozen tissue for double staining of HGF and Ym1, a marker of M2 macrophages. Each representative section was stained with hematoxylin-eosin, and immunohistochemical stains were performed with the avidin-biotin-peroxidase complex method^12,13,49^, using VECTASTAIN ABC kit (Vector Laboratories, Burlingame, CA). Antigen retrieval of paraffin section was achieved by a microwave, using the antigen retrieval solution (Dako, Carpinteria, CA). Non-specific serum blocking was performed by incubation with 5% goat serum for 30 min. Tissue sections were then incubated with primary antibodies at 4 °C overnight, followed by incubation with secondary antibodies at room temperature for 45 minutes. Tissue sections were then incubated with AB complex (VECTASTAIN ABC kit; Vector, Burlingame, CA) for 30 min according to the manufacture instruction to amplify signals, and reacted with DAB (SIGMAFAST 3,3′-Diamino-benzidine tablets, SIGMA Life Science, St Louis, MO, USA). Counterstaining was performed by using Haematoxylin for 20 seconds. The following antibodies were used: biotin conjugated anti-CD133 antibody (1:100; abcam 19898), anti-HGF antibody (1:200; abcam 837060), anti-SDF-1 antibody (1:200; abcam 25117), and biotinylated goat anti-rabbit IgG (1:20; Cell signaling). Double staining of HGF and Ym1 was also performed by immunofluorescent stains using frozen sections. HGF staining was carried out using anti-rabbit HGF antibody (1:200; abcam 837060) and FITC-conjugated donkey anti-rabbit IgG antibody (1:200; Jackson immunoreserch laboratories, West Grove, PA lot 712-095-153). After HGF staining, the sections were incubated with phycoerythrin (PE) conjugated anti-Ym1 antibody (1:200; abcam 211621) for 1 hour at room temperature. Cell nuclei were stained blue with DAPI. Tissue sections were analyzed by fluorescent microscopy.

### Statistical analysis

Quantitative data are expressed as mean ± standard deviation (SD). Adhesion scores were assessed using the one-way ANOVA test followed by Bonferroni-Holm posthoc tests for comparisons between four groups. Statistical analysis was performed using SPSS 13.0 software. P < 0.05 was considered significant.
